# Role of Biological Markers for Cerebral Bleeding Risk STRATification in Patients with Atrial Fibrillation on Oral Anticoagulants for Primary or Secondary Prevention of Ischemic Stroke (Strat-AF Study): Study Design and Methodology

**DOI:** 10.3390/medicina55100626

**Published:** 2019-09-23

**Authors:** Anna Poggesi, Carmen Barbato, Francesco Galmozzi, Eleonora Camilleri, Francesca Cesari, Stefano Chiti, Stefano Diciotti, Silvia Galora, Betti Giusti, Anna Maria Gori, Chiara Marzi, Anna Melone, Damiano Mistri, Francesca Pescini, Giovanni Pracucci, Valentina Rinnoci, Cristina Sarti, Enrico Fainardi, Rossella Marcucci, Emilia Salvadori

**Affiliations:** 1 Stroke Unit, Careggi University Hospital, 50134 Florence, Italy; francesca.pescini@unifi.it (F.P.); valentina.rinnoci@unifi.it (V.R.); cristina.sarti@unifi.it (C.S.); emilia.salvadori@unifi.it (E.S.); 2 NEUROFARBA Department, Neuroscience Section, University of Florence, 50134 Florence, Italy; carmenbarbato88@gmail.com (C.B.); galmo89@icloud.com (F.G.); melone.anna@hotmail.it (A.M.); damiano.mistri@gmail.com (D.M.); giovanni.pracucci@unifi.it (G.P.); 3 IRCCS Don Carlo Gnocchi, 50143 Florence, Italy; 4 Department of Experimental and Clinical Medicine, University of Florence, 50134 Florence, Italy; camillerieleonora@gmail.com (E.C.); silviagalora@gmail.com (S.G.); betti.giusti@unifi.it (B.G.); annamaria.gori@unifi.it (A.M.G.); rossella.marcucci@unifi.it (R.M.); 5 Central Laboratory, Careggi University Hospital, 50134 Florence, Italy; francesca.cesari@gmail.com; 6 Department Health Professions, U.O.c Research and Development, 50134 Careggi University Hospital, 50134 Florence, Italy; stefano.chiti@gmail.com; 7 Department of Electrical, Electronic, and Information Engineering “Guglielmo Marconi”, University of Bologna, 40136 Bologna, Italy; stefano.diciotti@unibo.it (S.D.); chiara.marzi3@unibo.it (C.M.); 8 Neuroradiology Unit, Department of Experimental and Clinical Biomedical Sciences, University of Florence, Careggi University Hospital, 50134 Florence, Italy; henryfai@tin.it

**Keywords:** atrial fibrillation, anticoagulation, stroke, intracerebral hemorrhage, cerebral small vessel disease, brain MRI, circulating biomarkers

## Abstract

*Background and Objectives:* In anticoagulated atrial fibrillation (AF) patients, the validity of models recommended for the stratification of the risk ratio between benefits and hemorrhage risk is limited. Cerebral small vessel disease (SVD) represents the pathologic substrate for primary intracerebral hemorrhage and ischemic stroke. We hypothesize that biological markers—both circulating and imaging-based—and their possible interaction, might improve the prediction of bleeding risk in AF patients under treatment with any type of oral anticoagulant. *Materials and* *Methods*: The Strat-AF study is an observational, prospective, single-center hospital-based study enrolling patients with AF, aged 65 years or older, and with no contraindications to magnetic resonance imaging (MRI), referring to Center of Thrombosis outpatient clinic of our University Hospital for the management of oral anticoagulation therapy. Recruited patients are evaluated by means of a comprehensive protocol, with clinical, cerebral MRI, and circulating biomarkers assessment at baseline and after 18 months. The main outcome is SVD progression—particularly microbleeds—as a selective surrogate marker of hemorrhagic complication. Stroke occurrence (ischemic or hemorrhagic) and the progression of functional, cognitive, and motor status will be evaluated as secondary outcomes. Circulating biomarkers may further improve predictive potentials. *Results*: Starting from September 2017, 194 patients (mean age 78.1 ± 6.7, range 65–97; 61% males) were enrolled. The type of AF was paroxysmal in 93 patients (48%), and persistent or permanent in the remaining patients. Concerning the type of oral anticoagulant, 57 patients (29%) were on vitamin K antagonists, and 137 (71%) were on direct oral anticoagulants. Follow-up clinical evaluation and brain MRI are ongoing. *Conclusions*: The Strat-AF study may be an essential step towards the exploration of the role of a combined clinical biomarker or multiple biomarker models in predicting stroke risk in AF, and might sustain the incorporation of such new markers in the existing stroke prediction schemes by the demonstration of a greater incremental value in predicting stroke risk and improvement in clinical outcomes in a cost-effective fashion.

## 1. Introduction

Thromboprophylaxis with oral anticoagulation effectively reduces stroke risk in patients with atrial fibrillation (AF). Benefits must be balanced against the risk of bleeding, with intracranial hemorrhage being the most feared. Stroke and bleeding risk stratification schemes are aimed at identifying patients who may benefit most from different types of oral anticoagulation (vitamin K antagonists vs. direct oral anticoagulants). Currently, such schemes (e.g., CHADS2VASC2 and HASBLED scores) rely only on clinical information, the validity of which remains controversial and needs to be improved. In AF patients, advanced imaging technology such as magnetic resonance imaging (MRI), has led to the increased detection of asymptomatic brain changes, mainly those related to small vessel disease (SVD), which is the pathologic substrate for primary intracerebral hemorrhage (ICH) [1,2,3,4,5,6]. These changes have also been proven to strongly predict stroke risk [2,5]. Such imaging findings, obtained both at baseline and in terms of lesion progression over time, could be used as additional markers for risk stratification [3,7]. Furthermore, markers of coagulation activation, including prothrombin fragment 1+2, thrombin–antithrombin complex, D-dimer, time in therapeutic range for warfarin, and drug dosage for new anticoagulants, may be also studied as cofactors. Preliminary data suggest that circulating biomarkers of endothelial dysfunction, hypercoagulable state, and inflammation may further enhance risk prediction [4].

The validity of the currently recommended models for the stratification of risk ratio between benefits and hemorrhagic risk in anticoagulated AF patients is limited. These models do not specifically take intracranial hemorrhage into account, which is indeed the most severe hemorrhagic complication [8]. Our hypothesis is that biological markers—both circulating and imaging-based, and their possible interaction—might improve the prediction of bleeding risk in AF patients treated with any type of oral anticoagulant. Neuroimaging biomarkers of SVD—particularly microbleeds—may serve as a selective surrogate marker of hemorrhagic complications. Circulating biomarkers assessed together with imaging may further improve predictive potentials.

In this scenario, we set up the prospective observational Strat-AF study, primarily aimed at investigating circulating biomarkers and MRI markers (baseline and progression) of SVD as surrogate markers for the prediction of cerebral bleeding in a cohort of patients with AF on oral anticoagulants. Secondary outcomes included stroke occurrence (either ischemic or hemorrhagic) and the progression of functional, cognitive, and motor status.

## 2. Materials and Methods

Stratification of Cerebral Bleeding Risk in AF, Strat-AF, Strat-AF is an observational, prospective, single-center hospital-based study enrolling patients with AF, referring to the Center of Thrombosis outpatient clinic of our University Hospital for the management of oral anticoagulation therapy.

### 2.1. Inclusion Criteria

- Diagnosis of AF and ongoing oral anticoagulation therapy;
- Aged ≥ 65 years;
- No contraindications to MRI.

### 2.2. Exclusion Criteria

- Inability or refusal to undergo cerebral MRI;
- Inability to give an informed consent.

All subjects gave their informed consent for inclusion before they participated in the study. The study was conducted in accordance with the Declaration of Helsinki, and the protocol was approved by the Ethics Committee of the Careggi University Hospital (Project identification code 16RFAP, approved on March 2017). 

Sample size was estimated based on a feasibility criterion (i.e., the real flow of patients to the Thrombosis outpatient clinic of Careggi Hospital). Yearly, approximately 600 patients with AF on oral anticoagulation refer to the outpatient clinic. Considering a foreseeable rate of ineligible patients and refusals, we originally estimated to contact approximately 300 patients in one year fulfilling the inclusion criteria. Starting from September 2017, consecutive eligible patients were invited to participate in the study. The initial foreseen period for enrollment was 12 months, but in order to augment the number of included patients, we extended the enrollment period to 18 months.

### 2.3. Clinical Assessment

All enrolled patients were assessed at baseline by means of a standard clinical/functional protocol collecting information on vascular risk factors (particularly hypertension and diabetes), dietary habits, previous cerebrovascular events, general neurological and functional status, cognitive performances, mood and gait disorders, and general neurological examination. 

In detail, clinical data were collected about social and medical history; a standard cardiovascular and neurological examination, including office blood pressure measurement, were performed. Functional status was assessed using the Activities of Daily Living scale and the Instrumental Activities of Daily Living scale [9,10]; mood assessment using the Geriatric Depression Scale [11]; motor performance using the Short Physical Performance Battery [12]; daytime sleepiness using the Epworth Sleepiness Scale [13]; and quality of life using the EuroQol Visual Analog Scale [14]. Dietary habits were assessed by means of the questionnaire on the adherence to the Mediterranean diet [15].

A comprehensive multi-domain cognitive assessment, including global functioning, orientation, memory, attention, executive functions, language, speed, and motor control was administered to each included patient. 

The cognitive domains assessed by means of the extensive battery of tests were as follows:

- Global cognitive efficiency, by means of the Montreal Cognitive Assessment (MoCA): it is a 10-minute cognitive screening tool created to detect mild cognitive impairment (MCI), suggested from the harmonization standards of the National Institute of Neurological Disorders and Stroke—Canadian Stroke Network (NINDS-CSN) and thought to be specifically sensitive to frontal, attention, and executive deficits [16,17,18]. It covers eight cognitive domains: short-term and delayed verbal memory; visuospatial abilities; executive functions; attention; concentration; working memory; language; and orientation (score range 0–30)
- Verbal memory, by means of the Rey Auditory-Verbal Learning Test (RAVLT) and the Short Story Recall Test [19,20]. The RAVLT and Short Story Recall Test measure several components of verbal memory, such as immediate free recall, verbal learning, and retention of information after a certain period of time. Two scores are obtained from the RAVLT: immediate free recall (range 0–75) and delayed free recall (range 0–15). The Short Story Recall Test has a total score obtained by the mean number of elements correctly remembered on the immediate and delayed recalls (score range 0–28).
- Attention, by means of the Visual Search test [21]. This is a number cancellation task that requires visual selectivity at fast speed, and assesses the capacity for sustained attention and accuracy of visual scanning (score range 0–60). The Colour Word Stroop test was also used [22]. It is a measure of concentration effectiveness and deals with response inhibition and selective attention. The activity required by this test is a selective processing of only one visual feature while continuously blocking out the processing of others. The execution time and the errors committed are recorded.
- Language, by means of:
  - Semantic verbal fluency test: a test allowing the semantic evaluation of lexical access [23]. Three categories were tested: car brands, fruits, and animals. The total score was given by the sum of the number of words produced for each category in one minute.
  - Sentence construction test: a test used to evaluate the verbal ability to construct a meaningful sentence starting from a set of two or three words (score range 0–25) [19].

All raw test scores were demographically corrected according to Italian population normative data, and adjusted scores were then recoded as normal, borderline, or abnormal according to equivalent scores (ES) methodology [24]. ES methodology is a non-parametric norming method based on percentiles and is independent from the distribution form. ES is an ordinal 5-point scale (ranging from 0 to 4), and the main point of ES methodology is to fix the outer tolerance limit of the left queue of the adjusted scores so that it is possible to assess, with a known risk of error (<5%), the cut-off splitting the bottom 5% of the population and representing pathological performance (ES = 0). At the other end of the scale, ES = 4 indicates an optimal performance (equal to or better than the median). ES = 1 indicates a borderline performance (an adjusted score between the outer and inner confidence limits for the fifth centile of the normal population), while the remaining ES scores of 2 and 3 represent normal performances.

The diagnosis of MCI requires at least one altered score (ES = 0) plus one borderline score (ES = 1) in any cognitive test included in the neuropsychological battery.

### 2.4. Cerebral Magnetic Resonance Imaging Assessment

Cerebral MRI scans were performed at baseline, and again 18 months after enrollment. The brain imaging protocol was planned and set up by imaging personnel with different expertise and skills, as suggested by current guidelines [25].

Baseline and follow-up MRI examinations are performed on an Ingenia 1.5-Tesla MRI unit (Philips Healthcare, Eindhoven, The Netherlands). Our standardized MRI protocol consisted of the following sequences: sagittal T1-weighted spin-echo (repetition time (TR) = 547 ms; echo time (TE) = 12 ms; slice thickness = 5 mm; interslice spacing = 0.5 mm; matrix size = 320 × 250; field of view (FOV) = 23 cm × 23 cm; number of signals averaged (NSA) = 1), coronal T2-weighted fast spin-echo (TR = 3347 ms; TE = 110 ms; slice thickness = 5 mm; interslice spacing = 0.5 mm; matrix size = 512 × 322; FOV = 22 cm × 22 cm; NSA = 2); axial fluid-attenuated inversion recovery (FLAIR) (TR = 11,000 ms; TE = 125 ms; inversion time (TI) = 2800 ms; slice thickness = 5 mm; interslice spacing = 0.5 mm; matrix size = 384 × 204; FOV = 23 cm × 23 cm; NSA = 2); axial gradient-echo T2* (GRE) (TR = 534 ms; TE = 23 ms; flip angle (FA) = 18; slice thickness = 5 mm; interslice spacing = 0.5 mm; matrix size = 256 × 185; FOV = 23 cm × 23 cm; NSA = 1); axial diffusion-weighted imaging (DWI) (TR = 3891 ms; TE = 75 ms; slice thickness = 5 mm; interslice spacing = 0.5 mm; matrix size = 164 × 162; FOV = 23 cm × 23 cm; NSA = 2); gradient-echo 3D T1-weighted (TR = 7.5 ms; TE = 3.4 ms; TI = 950, slice thickness = 1 mm; matrix size = 256 × 241; FOV = 25.6 cm × 25.6 cm; NSA = 1) followed by multiplanar reconstruction (MPR) in axial, coronal, and sagittal planes. DWI images were obtained by single-shot echo-planar spin-echo sequences according to the Stejskal–Tanner method. The diffusion gradients were applied in three orthogonal directions (*x*, *y*, *z*) with two b-values (0 and 1000 s/mm^2^) to form the isotropic DWI images at b 1000 s/mm^2^. Apparent diffusion coefficient (ADC) maps were generated automatically by the software provided by the manufacturer from isotropic DWI images and concurrent images with a b value of 0 s/mm^2^ by using the following equation: ADC = -ln [S(b)/S(0)]/b, where b indicates the b value and S(b) and S(0) are the signal intensities of images with b values equal to 1000 and 0, respectively.

Each MRI scan was evaluated by an expert neuroradiologist, and a written medical report was provided to patients. Neurologists involved in the project revised the report before returning it to patients, so that any incidental findings could be handled according to individual needs. 

Qualitative and quantitative analyses of SVD-related features on MRI are under way. 

The following features related to SVD will be assessed: i) lacunar infarcts, either silent or not; ii). white matter hyperintensities; iii) microbleeds; iv) dilated perivascular spaces; v) cortical and subcortical atrophy; vi) superficial siderosis [5].

Non-lacunar infarcts will be visually assessed in terms of number and location of lesions.

### 2.5. Circulating Biomarker Assessment

Venous blood samples were collected after enrollment and placed at -80 °C for long-term storage. 

Besides routine parameters (complete blood count (CBC), prothrombin time (PT), activated partial thromboplastin time (APTT), D-dimer, fibrinogen, low-density lipoprotein (LDL), high-density lipoprotein (HDL), triglycerides, glucose, creatinine, glomerular filtration rate (GFR), alanine aminotransferase, C-reactive protein (CRP), N Terminal pro B-type natriuretic peptide, Lp(a), troponin), several circulating biomarkers will be evaluated:

- Endothelial function biomarkers;
- Pro- and anti-inflammatory molecules;
- Metalloproteinases and their inhibitors;
- Markers of renal function;
- Markers of blood clotting activation and of fibrinolytic system of coagulation activation, including prothrombin fragment 1+2, thrombin–antithrombin complex, D-dimer, and endogenous thrombin potentials;
- Genetic polymorphisms that may influence the effect of anticoagulants and plasmatic microRNA profile.

The list of biomarkers under investigation is detailed in Table 1. The biological material (serum, plasma, DNA) is properly conserved to allow the evaluation of further biomarkers by specific or global assessment strategies (e.g., metabolomics profiling, targeted or whole-exome sequencing).

Proteomic and genetic biomarkers will be evaluated with high-multiplex immunoassays or high-throughput technologies such as Bio-Plex Multiplex Immunoassay System (BioRad), Proximity Extension Assay (Olink) technology, H-nuclear magnetic resonance spectroscopy (Bruker BioSpin), real-time PCR (Life Technologies), droplet digital PCR (BioRad), and high-throughput sequencing (Illumina).

Biosamples were processed using standard and harmonized operating procedures.

Clinical data are registered electronically in the web-based registry (http://www.strat-af.it/). Quality controls are done on a weekly basis. Imaging data are instantly checked for protocol conformity. 

The same clinical/functional assessment and brain MRI will be repeated 18 months after enrollment. An interim telephone follow-up interview will be scheduled approximately three months before the final clinical assessment. 

### 2.6. Primary Endpoint

The primary study endpoint will be SVD progression, evaluated by means of the control MRI performed 18 months after enrollment. The progression of lacunar infarcts and microbleeds will be evaluated as the appearance of at least one new lesion, respectively. White matter hyperintensities progression will be rated by means of the visual Rotterdam Progression Scale (score range from 0 to 9) [26]. Absence or presence of progression (0 or 1, respectively) will be rated in three periventricular regions (frontal caps, occipital caps, bands), four subcortical white matter regions (frontal, parietal, occipital, temporal), basal ganglia, and the infratentorial region. Post-processing and ratings will be centralized and performed by expert and reliable observers, blinded to clinical data.

### 2.7. Secondary Endpoints 

Secondary endpoints will be:

1. Stroke occurrence (ischemic or hemorrhagic).
2. Considering data from medical history and available laboratory and imaging exams, new major cerebrovascular events will be recorded as ischemic or hemorrhagic strokes. Ischemic strokes will be further categorized in subtypes according to the TOAST classification system [27]. Hemorrhagic strokes will be classified according to lesion type and location.
3. Progression of global functioning, cognitive, and motor performances.
4. Progression in cognitive status will be determined by the occurrence of a diagnosis of MCI or dementia, and defined according to performances on the comprehensive neuropsychological test battery (evaluated as normal or abnormal by means of national normative data), and functional status.
5. The change in global functional status will be based on the Activities of Daily Living and Instrumental Activities of Daily Living scales, and the worsened condition will be defined as the loss of at least one item (i.e., the patient became dependent for an item function that was preserved at baseline evaluation).
6. Motor status will be evaluated by means of the Short Physical Performance Battery. Based on the total score (range 0–12), at each visit individuals will be categorized as having normal (SPPB ≥ 11) or impaired mobility (SPPB ≤ 10), and variations in performance categories over time (baseline vs. 18 months) will be evaluated for each patient.

### 2.8. Statistical Analyses

Multivariate regression analyses will be performed in order to identify independent predictors of SVD and its progression. Among the variables of interest, apart from the clinical ones and those assessed by means of conventional MRI, special attention will be paid to the relationship with advanced neuroimaging features and circulating biological markers.

Cerebral SVD and its progression—particularly microbleeds, which are considered the main expression of a bleeding-prone state—will be studied as outcome variables in multivariate regression models, considering the independent effect of all relevant markers, including major vascular risk factors and anticoagulant treatment with different types of anticoagulants (vitamin K antagonists vs. direct thrombin antagonists or factor Xa antagonists), and circulating biomarkers. 

Interactions of circulating biomarkers with imaging markers, and types of oral anticoagulants will also be analyzed. Bonferroni correction will be applied in multivariate models to counteract the problem of multiple comparisons.

The multivariable prediction of the risk of SVD progression in this cohort of AF patients on oral anticoagulants will be studied as the methodological background for clinical research in the setting of stroke prevention in patients with AF (i.e., for designing and sample sizing studies potentially adopting the above indicated biomarkers as surrogate markers of the clinical outcomes).

## 3. Results

**Figure 1:**
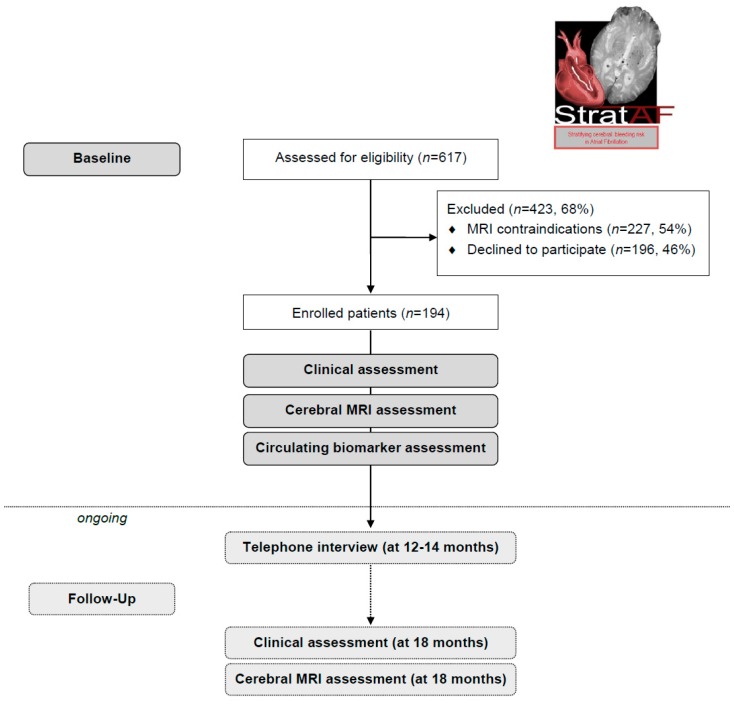
Strat-AF flow diagram.

Starting from September 2017 until March 2019, 617 patients referring to the outpatient clinic of the Center of Thrombosis for oral anticoagulants therapy control were screened for inclusion in the study. As shown in Figure 1, 423 patients (68%) were excluded because of MRI contraindications (*n* = 227) or refusal (*n* = 196). The remaining 194 patients were enrolled. Demographic and clinical characteristics of the baseline sample are shown in Table 2: mean age was 78.1 ± 6.7 (range 65–97) years, 118 (61%) were males, and mean education was 9.1 ± 4.3 (range 2–19) years. The type of AF was paroxysmal in 93 patients (48%), and persistent or permanent in the remaining patients. Concerning the type of oral anticoagulant, 57 patients (29%) were on vitamin K antagonists, and 137 (71%) were on direct oral anticoagulants. Follow-up clinical evaluation and brain MR are ongoing.

## 4. Discussion

The Strat-AF study is an ongoing, single-center, longitudinal observation study evaluating elderly patients with AF on oral anticoagulation for primary or secondary prevention of stroke. The project foresees, in consecutive elderly patients with AF attending the Center for Thrombosis outpatient clinic of Careggi University Hospital for management of oral anticoagulation therapy, the implementation of a comprehensive neurological, neuropsychological, and functional evaluation together with blood sample collection for the determination of circulating biological markers (in relation to the hemorrhagic risk profile) and brain MRI for the determination of brain parenchyma lesions as surrogate markers of a bleeding-prone state. The Strat-AF cohort will allow the evaluation of the possible role of biological markers, including clinical, circulating, and neuroimaging-based, and their interaction, on the prediction of bleeding risk in AF patients under treatment with any type of oral anticoagulant. Neuroimaging biomarkers of SVD, particularly microbleeds, will be tested as a selective surrogate marker of such complication. Circulating biomarkers assessed together with imaging might further improve the predictive potentials. Active enrollment started from September 2017 and ended in March 2019. The baseline study cohort included 194 elderly patients. Follow-up assessments are now ongoing. 

One first major comment arises from the fact that in order to reach the number of enrolled patients, more than 600 patients had to be screened. Of these, more than half were excluded. Main reasons for exclusion were refusal (46%) and MRI contraindications (54%). Thus, the method under evaluation (i.e., brain MRI) will not be feasible for all AF patients, but just for those without contraindication. According to our experience, about one-quarter of patients would not be included in such a new stratification risk schema.

Overall, the study protocol seems feasible, and nearly all included patients completed the evaluation.

Our results will provide a unique opportunity to achieve preliminary data about SVD progression as a surrogate marker of the effect of antithrombotic treatments used for stroke prevention in patients with AF. The longitudinal design of the study may also provide clues about the possible association of SVD and its progression with clinical endpoints. Thrombo-embolic and bleeding risk will be assessed using the clinical risk scores CHADS2VASC2 and HAS-BLED respectively. Such predictive models will be completed studying the effect of neuroimaging features, particularly those related to SVD, and circulating biological markers, in order to provide preliminary knowledge about the incremental value of such markers. 

Conclusive evidence about the predictive value of these markers in single patients can only come from adequately powered large prospective follow-up studies, and few efforts are already under way. Prospective multicenter studies such as the CROMIS 2 (clinical relevance of microbleeds in stroke; ClinicalTrials.gov Identifier NCT02513316), ICH because of oral anticoagulants: prediction of the risk by MRI (HERO, Hirulog Early Reperfusion/Occlusion Trial; ClinicalTrials.gov Identifier NCT02238470), and CMB-NOW (CMBs during NOACs or warfarin therapy in non-valvular AF patients with acute ischemic stroke; ClinicalTrials.gov Identifier NCT02356432) are ongoing. Main results from CROMIS-2 have been recently published: among the 1490 patients with AF enrolled after their ischemic stroke, the presence of CMBs significantly increased the risk of symptomatic ICH (adjusted hazard ratio 3.67, 95% CI 1.27–10.60), confirming that CMBs are independently associated with symptomatic ICH risk, and could be used to inform anti-coagulation decisions. In this study, after 24 months of follow-up, 14 patients had an intracerebral hemorrhage. The very low number of events did not allow the establishment of whether a CMBs threshold exists. Moreover, the study does not foresee a control MRI, so no information is available concerning the possible role of CMBs progression. A recent meta-analysis assessed the association between CMBs and future ICH risk in ischemic stroke patients with AF taking oral anticoagulants. The authors concluded that the presence of CMBs on MRI and the dichotomized cutoff of ≥5 CMBs might identify subgroups of patients with high ICH risk [28], but these data need to be confirmed before they can be used in clinical practice. 

## 5. Conclusions

Long-term oral anticoagulation is the mainstay therapy for ischemic stroke prevention in patients with AF. Available stroke and bleeding risk stratification schemes are aimed at identifying patients who may benefit most from oral anticoagulation [29]. Such schemes (e.g., CHADS2VASC2, HAS-BLED scores) currently rely only on clinical information, the validity of which remains controversial and needs to be improved. Attempts have been made to refine the risk stratification scores by the addition of various biomarkers (blood, urine, cardiac, and cerebral imaging), but data are still inconclusive as to whether the costs are justified [30]. 

The Strat-AF study may be an essential step towards the exploration of the role of a combined clinical biomarker or multiple biomarker models in predicting stroke risk in AF, and might sustain the incorporation of such new markers in the existing stroke prediction schemes by the demonstration of a greater incremental value in predicting stroke risk and improvement in clinical outcomes in a cost-effective fashion.

## Figures and Tables

**Table 1:** Circulating biomarkers under investigation in the Strat-AF study.

Category	Biomarkers Name
Markers of endothelial function	- von Willebrand Factor (vWF) - Tissue Factor Pathway Inhibitor (TFPI)
Pro- and anti-inflammatory molecules	- Chemokine (C-C motif) ligand 3 (CCL3) - Interleukin (IL)-1β (IL-1β), - IL-1 Receptor antagonist (IL-1RA) - IL-2 - IL-6 - IL-8 - IL-10 - IL-12 P40 - Tumor Necrosis Factor Alpha (TNF-Alfa) - Intercellular Adhesion Molecule-1 (ICAM-1) - Vascular cell adhesion molecule-1 (VCAM-1) - Vascular Endothelial Factor (VEGF)
Metalloproteinases	- Matrix metalloproteinase (MMP)-2, MMP-8, MMP-9, MMP-12 - Tissue Inhibitor of Metalloproteinases (TIMP)-1, TIMP-2, TIMP-3, TIMP-4
Markers of coagulation activation	- Endogenous thrombin potential (ETP) - Plasminogen activator inhibitor-1 (PAI-1) antigen - Prothrombin fragment 1+2 (F1+2) - Thrombin antithrombin complexes (TAT) - Tissue factor - Plasminogen Activator Inhibitor-1 (PAI-1) antigen - Clot lysis time (CLT)
Genetic polymorphisms and plasmatic miRNA	- CES1 rs2244613 - CES1 rs8192935 - ABCB1 rs148738 - VKORC1 G3673A or –1639G>A rs9923231 - CYP2C9*2 rs1799853 - CYP2C9*3 rs1057910 - CYP2C9*5 rs28371686 - CYP2C9*6 rs9332131 - CYP4F2 V433M rs2108622 C>T - GGCX rs11676382 - Plasmatic miRNA profiling by real-time PCR

**Table 2:** Baseline sample demographic and clinical characteristics.

	Min–Max	Baseline Sample *n* = 194
Age, years (mean ± SD)	65–97	78.1 ± 6.7
Years of education	2–19	9.1 ± 4.3
Sex (% males)	-	61% (*n* = 118)
Hypertension	-	82% (*n* = 159)
Hypercholesterolemia	-	48% (*n* = 94)
Diabetes	-	13% (*n* = 25)
Smoking habits	-	61% (*n* = 119)
History of stroke	-	22% (*n* = 42)
Alcohol consumption	-	51% (*n* = 100)
Paroxysmal AF	-	48% (*n* = 93)
Type of oral anticoagulant Vitamin K antagonists Direct oral anticoagulants	- -	29% (*n* = 57) 71% (*n* = 137)
